# COVID-19 Detection by Means of ECG, Voice, and X-ray Computerized Systems: A Review

**DOI:** 10.3390/bioengineering10020198

**Published:** 2023-02-03

**Authors:** Pedro Ribeiro, João Alexandre Lobo Marques, Pedro Miguel Rodrigues

**Affiliations:** 1 CBQF—Centro de Biotecnologia e Química Fina—Laboratório Associado, Escola Superior de Biotecnologia, Universidade Católica Portuguesa, Rua de Diogo Botelho 1327, 4169-005 Porto, Portugal; 2 Laboratory of Applied Neurosciences, University of Saint Joseph, Macao SAR 999078, China

**Keywords:** COVID-19, artificial intelligence, signal processing, image processing, computerized diagnostic systems

## Abstract

Since the beginning of 2020, Coronavirus Disease 19 (COVID-19) has attracted the attention of the World Health Organization (WHO). This paper looks into the infection mechanism, patient symptoms, and laboratory diagnosis, followed by an extensive assessment of different technologies and computerized models (based on Electrocardiographic signals (ECG), Voice, and X-ray techniques) proposed as a diagnostic tool for the accurate detection of COVID-19. The found papers showed high accuracy rate results, ranging between 85.70% and 100%, and F1-Scores from 89.52% to 100%. With this state-of-the-art, we concluded that the models proposed for the detection of COVID-19 already have significant results, but the area still has room for improvement, given the vast symptomatology and the better comprehension of individuals’ evolution of the disease.

## 1. Introduction

The World Health Organization (WHO) has been on alert since early 2020 regarding the Coronavirus Disease 19 (COVID-19). Nowadays, with well over 6 million deaths worldwide [1], the scientific community is developing new ways to detect the disease.

### 1.1. Mechanism

COVID-19 is a disease caused by a Severe Acute Respiratory Syndrome-Coronavirus-2 (SARS-CoV-2) [2], which is a single, positive-strand Ribonucleic acid (RNA) virus that causes severe respiratory syndrome in humans. SARS-CoV-2 belongs to the family Coronaviridae and is divided into alpha (α-CoV), beta (β-CoV), gamma (γ-CoV), and delta (δ-CoV) coronaviruses. It was initially detected in bats, and the first cases of the disease were detected in a market in China. For this particular case, SARS-CoV-2 is a coronavirus genetically similar to β-CoV which, similar to α-CoV, can infect mammals [3,4,5].

SARS-CoV-2 uses Angiotensin Converting Enzyme 2 (ACE2), which is a receptor in the cell surface, to start the infection. After the binding of the spike protein with the ACE2 receptor, the invasion process is triggered by host cell proteases. The virus releases the RNA into the host cell, then the RNA is translated into viral replicase polyproteins. The negative RNA copies of the viral genome are produced by the enzyme replicase using the positive RNA genome. During transcription, RNA polymerase produces a series of subgenomic mRNAs and translates them into viral proteins. The RNA genome is assembled into virions in Golgi and Endoplasmic Reticulum (ER), which bud into the ERGIC (ER–Golgi intermediate compartment) and are released out of the cell [3,4,5].

SARS-CoV-2 uses ACE2 to initiate the infection process. This receptor is present in the kidney, blood vessels, heart, and the lungs, which means it can cause respiratory, cardiovascular, gastrointestinal, and central nervous system diseases [3,4,5].

In the next section, the most frequent symptoms identified in COVID-19 patients, as well as eventual complications, are briefly presented.

### 1.2. Symptoms

COVID-19 patients may present mild to severe symptoms, with a substantial portion of the population not demonstrating any type of symptoms. The reported symptoms include fever, cough, and shortness of breath. A small segment of the population presented some gastrointestinal symptoms such as vomiting, diarrhea, and pain in the abdominal area [4,5].

Cardiovascular complications have been reported in COVID-19 patients as well. The reports have described acute cardiac injury, cardiogenic shock, electrocardiographic (ECG) changes, right ventricular dysfunction, thromboembolic complications, and tachyarrhythmias [6].

### 1.3. Laboratory Diagnostic

Diagnosing active cases of COVID-19 is one of the most important tasks for controlling the pandemic. Laboratory testing techniques have been developed to obtain an accurate diagnosis of COVID-19. The most common techniques are Nucleic Acid Amplification Test (NAAT) and Antigen detection [4,7].

NAAT is a technology used to diagnose an active COVID-19 infection by the use of Real-Time Polymerase Chain Reaction (RT-PCR) assay to detect SARS-CoV-2 RNA from the upper respiratory tract [4,7].

Antigen detection tests are tests used to detect the presence of SARS-CoV-2 viral proteins. Most of the available antigen kits require samples taken from the nasal cavity or nasopharynx, with some kits allowing samples from saliva as well [7].

### 1.4. Rational for the Review

To determine the most suitable computerized resources to detect COVID-19, we investigated the scientific literature available up to November 2022. A research study was conducted in the Cochrane reviews database and in the PubMed/MEDLINE database to find the most up to date pieces of evidence and guidelines. We have read and analyzed the results based on the most popular indicators for evaluating classifiers algorithms, such as Accuracy, F1-Score, Sensitivity, and Specificity. With these metrics, it is possible to compare the proposed models in the literature and how they could possibly distinguish COVID-19 patients from other types of diseases or healthy control groups.

### 1.5. Objectives/Questions for the Review to Address

This review will analyze and compare a variety of computerized systems that detect COVID-19, with the main goal of determining if it is possible to accurately detect COVID-19 without the need for an RT-PCR test, preferably considering noninvasive methods.

## 2. Methods

### 2.1. Document Search

The document search was done using Google Scholar as the electronic search engine and was based on available literature from the databases Elsevier, Wiley, Knowledge E, Frontiers Media, SBMU Journals, Jaypee Brothers Medical Publishing, Springer Science and Business Media, MDPI, IEEE, Cold Spring Harbor Laboratory, Tech Science Press, and arXiv. The articles were accepted if they had at least two participant groups: COVID-19 patients and a Control group, and provided at least the metric Accuracy of their model.

The document search was performed between April 2022 and November 2022. The search keywords used were “COVID-19 AI Detection”, “COVID-19”, “COVID-19 Detection”, “COVID-19 detection ECG”, “COVID-19 detection X-ray”, “COVID-19 detection Voice”, “COVID-19 detection ECG Accuracy”, “COVID-19 detection X-ray Accuracy”, “COVID-19 detection Voice Accuracy”, “COVID-19 WHO”, “COVID-19 Heart Variable Rate”, “COVID-19 Image processing”, “COVID-19 Voice analyzes”, “COVID-19 cough detection”, “COVID-19 signal processing”, “COVID-19 Accuracy”, “COVID-19 computerized system”, and “COVID-19 breathing detection”.

### 2.2. Search Strategy

The article search process was initialized by searching the Keyword “COVID-19” on Google Scholar. Due to the recent appearance of COVID-19 we limited the search between the years 2020 and 2022, resulting in 483,000 papers. Later, we searched for the keywords “COVID-19 Detection”, resulting in 12,000 papers.

For the next step of our search, we took into consideration which parts of the body are the most affected by the disease and searched for the most common biomedical signals/images. We selected X-ray, Voice, and ECG as the biomedical signal/image sources and searched for the keywords “COVID-19 detection ECG” (950 papers), “COVID-19 detection Voice” (2100 papers), and “COVID-19 detection X-ray” (6300 papers).

As the inclusion criterion, we required that the articles must at least use Accuracy as a classification metric. For that we added the keyword “Accuracy” to the previous search, resulting in 855 papers with keywords “COVID-19 detection ECG Accuracy”, 1750 papers for the search “COVID-19 detection Voice Accuracy” and 6020 papers for the search “COVID-19 detection X-ray Accuracy”.

The exclusion criteria used were to discard state-of-the-art, reviews, systematic reviews, duplicate paper, and irrelevant abstracts, giving us a total of 550 papers.

Of the 550 papers for full-text analysis, 1 paper was removed because it was retracted and 249 papers were rejected because they did not allow public access to the database, did not give an exact number of samples present in the database, or did not indicate the number of samples used for training/testing.

In the final step of our search, 280 papers were removed because they did not provide a code or the applied process was not explained well enough for us to reproduce the proposed methodology. In the end, 20 papers were included in this review.

### 2.3. Limitations

Our review presents some limitations on the methods applied to obtain the references. For example, the use of Google Scholar as a search engine, although being capable of indicating papers from a variety of different publishers or even being capably of analyzing a full paper, its searching proprieties can present some limitations, e.g., the way we can apply the exclusion criteria, if we ask the search engine to discard state-of-the-art, reviews, and systematic reviews, it may exclude some original research articles because, e.g., “state-of-art” is included in those papers and it is an exclusion keyword. This type of limitation can remove several papers from the search that may present some interesting findings.

Due to the purpose of the present review being concerned with COVID-19 detection by using computerized systems, the code used and classification model are the most important information to provide. With that in mind, another limitation was the lack of documentation or even the sharing of algorithms to make the proposed methodology reproducible, which led to the exclusion of papers that did not provide any way for us to reproduce and fully understand the methodology.

**Figure 1:**
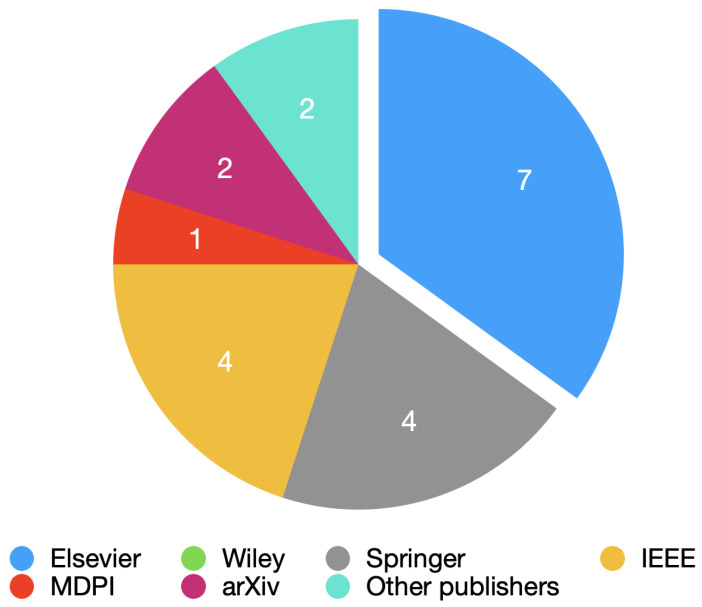
Pie chart with all the publishers used.

Figure 1 shows the number of papers per publisher obtained for this review.

### 2.4. Year of Publication Present in the Review

**Figure 2:**
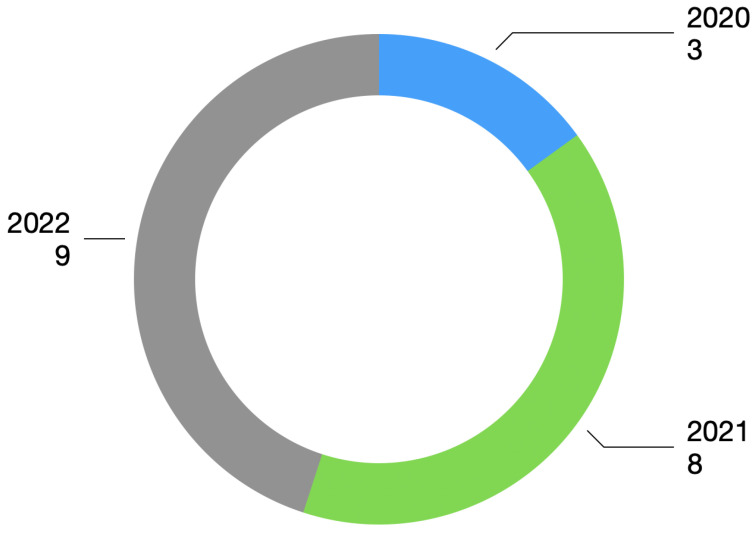
Year of publication.

The publication interval considered papers published between 2020 and 2022. Figure 2 shows the number of selected papers for each year.

The papers used in this review investigated the use of three different computerized systems: Voice processing, cardiovascular analysis based on Electrocardiogram (ECG) signal, and pulmonary assessment based on X-ray images.

## 3. Results

### 3.1. COVID-19 Detection Based on ECG Processing

Most of the impact of COVID-19 is focused on the respiratory system, but the virus can also cause a variety of cardiac complications, including myocardial injury, heart failure, cardiogenic shock, and cardiac arrhythmias, which shows the importance of ECG [8,9].

ECG is an exam that can monitor the electrical activity of the heart. In the early cases of COVID-19, myocardial Injury was found in patients that were infected with the virus [10].

As one of the most used clinical examination methods, it is of great importance to study the changes in the electrocardiographic activity, as well as to understand the ECG features related to COVID-19 [11].

In a study done in 2020 by Bergamaschi et al. [10], 269 patients were admitted with COVID-19. The ECGs were made at the admission date and after 1 week from hospitalization. The authors evaluated the correlation between ECGs findings and major adverse events (MAE). The study concluded that abnormal ECG at hospitalization and elevated baseline Troponin values were more common in patients who developed MAE. Other studies [11,12,13,14,15] concluded that Troponin is a good indicator to access the severity of the infection and the ECG might be an easy tool for risk stratification in such patients. In the same year, another study done by Angeli et al. [16] concluded that the evolution of ECG abnormalities is independent of the severity of pulmonary tract infection and reflects a wide spectrum of cardiovascular complications.

Looking into the ECG abnormalities, several studies found that the S-T segment alteration was the most frequent ECG finding and signs of left ventricular hypertrophy were associated with a worse prognosis [2,11,14,15], concluding that abnormal T wave or the presence of S-T segment elevation/depression can have a good prognostic in predicting the mortality of COVID-19 patients [11,14,15].

A study carried out by Bassiouni et al. [17] created several deep learning models and classifiers to distinguish COVID-19 from other cardiovascular diseases (CVDs) and Control, having the best Accuracy result of 99.74% with the ECGConvnet being used as a classifier. The ECGConvnet was the proposed system used in this study and it demonstrated that it is possible to develop an automatic diagnosis system for COVID-19 based on deep learning using ECG images.

A study [18] done in 2022 aimed to automatically utilize ECG signals to detect COVID-19. The ECG signal was obtained from ECG paper records, then the electrocardiographic signal was entered as input into a one-dimensional convolutional neural network (1D-CNN), and the authors tried to correctly diagnose the pathologies present in the database. The investigators separated the database into three different classes: COVID-19, Normal, and Other. The Other class contained the diseases myocardial infarction (MI), abnormal heartbeats, and recovered myocardial infarction (RMI). The investigation obtained an Accuracy of 83.17%, an F1-score of 85.38%, a Sensitivity of 84.81%, and a Specificity of 86.28% when using the three classes at the same time as the target.

Another study [19] submitted in 2022 approached the automatic detection of COVID-19 by utilizing models of Convolutional Neural Networks (CNN). The investigators tested the CNN pre-trained models ResNet50, DenseNet-201, VGG16, VGG19, Inceptionv3, and Inceptionresnetv2. ECG pre-processing was performed to eliminate undesirable distortions. Then, a data augmentation technique was implemented as a way to artificially inflate the dataset before entering the CNN models, and from all the models tested in this study, the VGG16 model had the best result of Accuracy with 81.39% for a target containing Normal ECGs and COVID-19 patients.

Attallah [20] investigated the use of Bi-Layers of deep features integration to diagnose COVID-19 based on ECG images. The paper used a methodology with four stages: preprocessing, feature extraction and integration, feature selection, and classification. The features were extracted from the last average pooling layer and the last fully connected layer from some pre-trained CNNs, which were the ResNet-50, the DenseNet-201, the Inception-V3, Xception, and the Inception-ResNet. The study concluded with 98.80% Accuracy, 98.8% Specificity, and a Sensitivity of 98.8% when doing a Binary classification between Normal ECGs and COVID-19 ECGs. The Multi-class Classification, which was the same class as the Binary plus Abnormal ECGs, had 91.73% Accuracy, 91.80% F1-Score, 95.9% Specificity, and 91.7% Sensitivity.

Sobahi et al. [21] published an article in 2022 that demonstrated an ECG-based COVID-19 detection. The investigators approached the situation with the use of an attention-based 3D CNN model with residual connections (RC). The database that was used contained 12-lead ECG printouts and was distributed between three classes: normal subjects, COVID-19 patients, and patients with abnormal heartbeat (AHB). The CNN model was comprised of 19 layers: 1 image 3D input, 3 3D convolution layers, 3 batch normalization layers, 3 rectified linear unit (ReLu) layers, 2 dropout layers, 2 additional layers, 1 Sigmoid layer, 1 Elementwise Multiplication layer, a fully connected layer, a softmax and classification layers. The study concluded with a Binary Classification (COVID-19 patients vs. Normal subjects) Accuracy of 99% and a Multiclass Classification (Covid patients vs. Normal subjects vs. Abnormal Heartbeat patients) Accuracy of 92%.

An investigation [22] published in 2022 considered a public dataset containing ECG images to diagnose COVID-19. Inside the database, there were five distinct categories, such as normal, COVID-19, MI, AHB, and RMI. They tested six different CNN models as a way to distinguish COVID-19 from the other types of classes. The models were ResNet18, ResNet50, ResNet101, InceptionV3, DenseNet201, and MobileNetv2. The investigators used six different classes: normal, COVID-19, MI, AHB, RMI, and CVDs. They also visualized three different classification schemes: a Binary classification between the normal class and the COVID-19 class, a three-class classification between the normal class, the COVID-19 class, and the CVDs class, and a five-class classification between the normal class, the COVID-19 class, the MI class, the AHB class, and the RMI class. For the Binary classification, the best result was 99.1% Accuracy, for the three-class classification the best Accuracy result was 97.36%, and for the five-class classification the best Accuracy was 97.83%.

Even though COVID-19 is, for the most part, a respiratory or lung disease, the cardiac system can also suffer significant damage. One common complaint of COVID-19 patients is the appearance of palpitations or even the rise of symptoms similar to a heart attack, which includes chest pain, shortness of breath, and Echocardiogram changes [23].

A non-invasive method such as the biomarker Heart Rate Variability (HRV) is a way to assess the Autonomic Nervous System (ANS) activity as an interaction between the respiratory, cardiovascular, and nervous systems, which means that it can be another possible way of studying the difference between COVID-19 and non-COVID-19 patients [24].

A study done by Mishra et al. [25], in 2020 took advantage of the heart rate sensors present on wearable devices. The authors found that elevated resting heart rates and outlying HR/steps measurements were altered, usually in advance of the symptoms.

A study [26] published in 2021 used a methodology in which the data was collected through a smartphone camera using photoplethysmography technology, wrist-worn smartwatches, and wrist-worn bands synchronized with a smartphone app. The investigators used three different classes: Before COVID-19, during COVID-19, and after COVID-19 for patients that used the smartphone app and were positive for the disease. They concluded that there was no statistically significant interaction between the HRV indicators before, during, and after COVID-19 illness. However, they found statistical differences in the standard deviation of normal-to-normal intervals (SDNN) and root mean square of successive normal-to-normal interval differences (RMSSD) for some patients.

Another study done by Hasty et al. [27] compared the levels of C-reactive protein (CRP), which is a marker of systemic inflammation, associated with severe disease in bacterial or viral infections [28], with the SDNN. In this experiment, they used patients that presented hypoxic respiratory failure requiring high-flow nasal cannula or mechanical ventilation, and the experiment was done for seven days. The study concluded that there was a drop of more than 40% in the standard deviation of the interval between heartbeats (SDNN) followed by more than a tripling of CRP in the 72 h that followed.

### 3.2. COVID-19 Detection Based on Voice Processing

Voice can be diagnosed and analyzed to determine the presence of a respiratory disease [29].

Let us take a look at the use of speech for disease detection. Speech is a complex process that requires the coordination of the brain, muscles, and respiratory system. The smallest changes in a person’s speech may be the early signs of a disease, for example, a disease such as Parkinson’s, which can be associated with tremors of the vocal cords [30].

One of the focal areas for COVID-19 is the lungs. The virus can cause lung complications such as pneumonia or even acute respiratory distress syndrome. For example, the use of systems to detect the slight changes in our voices that we humans are unable to hear is extremely important to detect a pathology that can provoke breathing difficulties [31].

Voice signal as a way to detect COVID-19 might be used not only with speech but also with coughing. Cough detection can be used to differentiate coughing sounds, and the coughing produced by COVID-19 is possibly one of the ways to go for the detection of the pathology [32,33,34].

An article [35] published in 2022 demonstrated the possibility of detecting COVID-19 through coughing. In this study, the researchers used four different classes: COVID-19 positive, COVID-19 negative, non-COVID-19 subjects, and non-COVID subjects with pertussis cough. The study demonstrated the feasibility of the automatic diagnosis of COVID-19 from coughs with an Accuracy, F1-Score, Specificity, and Sensitivity close to 90%, using Random Forest as the classifier.

Another study [36] from 2021 investigated the use of symbolic recurrence quantification measures with MFCC features for the automatic detection of COVID-19 in cough sounds of healthy and sick individuals. The investigators used the XGBoost as the classifier and the results obtained by the created model achieved an Accuracy of 99% with an F1-Score of 69%, for sustained vowels.

A study carried out by Dash et al. [37] developed a new feature that they called COVID-19 Coefficient (C-19CC). In speech recognition, the normal frequency scale to the perceptual frequency scale and the frequency range of the filter values are fixed. The characteristics of speech signals vary from disease to disease. In the case of the detection of COVID-19, mainly the coughing sounds, the bandwidth, and properties are quite different from the complete speech signal. The Accuracy result for C-19CC was 85.70% while using SVM to classify coughing sounds.

Atmaja et al. [38] submitted a paper in 2022 related to COVID-19 detection through coughing. The investigators proposed a transfer learning approach as a way to improve the performance of COVID-19 detection by incorporating cough detection, cough segmentation, and data augmentation. Cough detection was used to remove non-cough signals. Cough segmentation was used to segregate several coughs in a waveform into individual coughs and data augmentation was used to increase the number of samples used for deep learning. The investigators used three different datasets, Coswara, COUGHVID, and ComParE-CCS, having a total of 2026 samples, after cough detection and cough segmentation. The study used the Mel spectrogram to get the feature of the acoustic signal. The study concluded with an Accuracy of 88.19% using the CNN14 as a classifier, which is a Convolutional Neural Network that has 14 layers between the input layer and the output layer.

In 2020, a study done by Imran et al. [39] compared different types of cough and used artificial intelligence to distinguish patients with COVID-19 and patients without COVID-19. The study concluded that it is possible to create an app that can accurately distinguish COVID-19 and non COVID-19 patients by using Deep Transfer Learning-based Multi Class classifier, having an Accuracy of 92.64%.

Verde et al. [40] compared the performance of some machine learning techniques to correctly detect COVID-19 by analyzing the voice. The study, published in 2021, used a crowd-sourced database named Coswara, which is a database present on the GitHub platform and contains samples of coughing, breathing, and voice sounds from each subject. The investigators evaluated the sustained phonation of the vowels “a”, “e”, and “o”, because it avoided any linguistic artifacts due to the different languages present in the database. The features that were extracted from the voice samples were Fundamental Frequency (F^0^), Jitter and Shimmer, Harmonic to Noise Ratio (HNR), Mel-Frequency Cepstral Coefficients (MFCC), First and second derivatives of cepstral coefficient, Spectral Centroid (SC), and Spectral Roll-off (SR). The Machine Learning techniques used were divided into several groups, which were Bayes, Functions, Lazy, Meta, Rules, and Trees. The investigation concluded with the SVM Algorithm, present in the Machine Learning Functions, having the best overall result, obtaining an Accuracy of 97.07%, an F1-score of 82.35%, and a Specificity of 97.37%.

Silva et al. [41] used the Coswara dataset to extract features, such as Energy, Entropies, Correlation Dimension, Detrended Fluctuation Analysis, Lyapunov Exponent, and Fractal Dimensions, in a multi-band analysis done by Wavelet Transform. After the extraction, a feature selection was made and the selected features served as entries for an ensemble machine learning model (XGBoost). The classification results presented accuracies higher than 83%, obtained for all Binary pairs, with a special mention to the pair Healthy control vs. all stages of COVID-19, which had been discriminated with an Accuracy of 98.46%.

### 3.3. COVID-19 Detection Based on Image Processing

Image processing is the process of obtaining visible images of the inner body structure. The goal of this process is its use for scientific and medicinal purposes, as well as, tissue visual representation [42].

The use of Image processing is an interesting way to approach the diagnosis of COVID-19 because of the virus nature. The pathology can cause damage to the lungs and with the use of equipment such as Computed Tomography (CT) Scanners that can create an image of the affected organ, it can be used to complement the already existing diagnosis exams [43].

A research done by Salman et al. [44] aimed to construct a model by using deep learning tools for detecting COVID-19 pneumonia on high-resolution X-rays. The investigators used a CNN InceptionV3 as the classifier and obtained an Accuracy of 100%, which is a comparable performance against expert radiologists.

In another study conducted by DeGrave et al. [45], the researchers used Artificial intelligence (AI) to demonstrate that deep learning systems can detect COVID-19 from chest radiographs. They concluded that the deep learning models rely on confounding factors rather than medical pathology, which means that the systems appear accurate with the used dataset but failed when tested with new data.

In 2021, an investigation was conducted using AI and X-rays images. Öztaş et al. [46] compared the detection of COVID-19 between X-ray images and Blood test data. The study used ResNet-18 and squeezenet as training models to classify the images and a multi-layer neural network to diagnose the blood test. The researchers concluded that the ResNet-18 performs slightly better than squeezenet even though both have obtained almost 98% Accuracy. When comparing the X-ray methodology to the blood test methodology, The radio-graphic images performed better, having an Accuracy of almost 98%, compared to the 72% Accuracy obtained using the multi-layer neural network for the blood test.

Wu et al. [47] published an article about the use of deep Convolutional Neural Networks (CNNs) to diagnose positive COVID-19 cases, using X-ray images as their input. The proposed architectures for the CNN followed a simple LeNet-5, where the two structures were *in_6c_2p_12c_2p* and *in_8c_2p_16c_2p*. Each structure had the number of input nodes and c or p for the type of layers, which in this case were the convolution and pooling layers, respectively. This article obtained a final Accuracy of 98.83%, demonstrating that this can be another possible research direction.

Another study [48], also done with X-ray images, illustrated an automated diagnosis model from a dataset of X-ray images of patients with severe bacterial pneumonia, reported COVID-19 disease, and normal cases. The findings in this article indicate that deep learning with X-ray imagery could retrieve important biomarkers relevant for COVID-19 disease detection, obtaining a 96.73% of Accuracy on a modified ResNet-18.

Using the same type of images as the previously referred articles related to Image Processing, a paper [49] was done where the researchers used a methodology based on the deep feature plus support vector machine (SVM). The dataset was separated into three categories: COVID-19, pneumonia, and normal. The highest Accuracy was 98.66%, achieved by a combination of ResNet50 plus SVM.

Fang et al. [50] investigated the use of classifiers to identify positive cases of COVID-19. The article that was published in 2021, used chest x-ray images as input for the classifiers and they proposed a multi-stage residual network, named MSRCovXNet, for effective detection of the pathology. The investigators also used ResNet-18 as the feature extractor. The proposed network was optimized by fusing two feature enhancement modules, one containing local information and the other containing semantic information. The network obtained a precision of 98.90% for the detection of COVID-19 and, when using the COVIDGR dataset as the input, an average Accuracy of 82.20% was achieved.

An article [51] published in 2020 proposed an alternative method to determine the COVID-19 cases from normal or abnormal cases by using X-ray images. The investigators proposed the use of an enhanced cuckoo search optimization algorithm (CS) using fractional-order calculus (FO) and four different heavy-tailed distributions: the Mittag-Leffler distribution, Cauchy distribution, Pareto distribution, and Weibull distribution. The classification, done by using a KNN model, contained three classes: normal patients, COVID-19-infected patients, and pneumonia patients. The experiment used 18 different datasets and the best Accuracy result was 100%.

Table 1 indicates the strategy used in state-of-the-art methods to diagnose COVID-19 and Table 2 presents the discrimination rates of the previously presented methods.

## 4. Discussion

The main objective of this review was to answer the following research question: is it possible to accurately detect COVID-19 without the need for an RT-PCR test, preferably considering noninvasive methods?

### 4.1. ECG Processing

Looking into Table 2, in the articles related to the ECG [17,18,19,20,21,22], we can see high Accuracy results on all articles, with the lowest percentage being 81.39% and the highest percentage being 99.74%. The majority of the articles presented four discrimination metrics and just one provided Accuracy as the only metric.

Refs. [17,18,20,21,22] presented the metric F1-score. In these articles, we saw values ranging from 85.38% to 99.70%, which means that some models could correctly predict all the classes or had slight difficulty detecting some classes.

Of all the articles that had four metrics, the article by Nguyen et al. had the least favorable results, with Accuracy presenting 83.17% as the lowest percentage and Specificity obtained 86.28% as the highest result. These results demonstrated that some classes were not classified correctly.

### 4.2. Voice Processing

Looking into Table 2, in the articles related to the Voice signal [35,36,37,38,39,40,41], three articles presented Accuracy as the only metric. In those articles, the results of Accuracy were 85.70% and 98.46%, demonstrating that they have high Accuracy but are not able to correctly detect all the predicted classes.

The other three articles presented the Accuracy and the F1-score. The first article presented an Accuracy of 99.00% and an F1-score of 69.00%. The second article showed an Accuracy of 92.64% and an F1-score of 92.66%. The third article presented an Accuracy of 97.07% and an F1-score of 82.35%. The three articles presented high Accuracy percentage results but the first article demonstrated the lowest F1-Score result, indicating a low recall and precision, which is confirmed by the Sensitivity of 70.00%.

Three articles presented the four metrics, 85.53% Accuracy, 85.58% F1-score, 85.96% Sensitivity, 85.09% Specificity, 92.64% of Accuracy, 92.66% F1-score, 92.64% Sensitivity and 97.55% Specificity and 97.07% Accuracy, 82.35% F1-score, 93.33% Sensitivity and 97.37% Specificity, respectively. By looking into the four metrics at the same time, we can see high results for all the metrics but the articles done by Tena et al. had an overall higher difficulty to classify some tests.

### 4.3. X-ray Processing

Regarding the articles related to X-ray [44,46,47,48,49,50,51] presented in Table 2, five articles showed Accuracy as the only metric to evaluate the models. The Accuracy results ranged between 82.2% and 100%, which shows, in some models, difficulty in correctly predicting the classes.

The article by Sethy et al. showed three additional metrics apart from Accuracy. The results showed 95.38% Accuracy, 95.52% F1-score, 97.29% Sensitivity, and 93.47% Specificity. With this high percentage, we can see the false positive having a greater impact, making the F1-Score and the Specificity have a lower percentage when compared to the Accuracy.

Salman et al. used a model that gave us four metrics: Accuracy, F1-Score, Sensitivity, and Specificity. The results were 100% in all metrics, which shows that the model can predict all classes without difficulties. However, by combining training, validation, and testing there was a total of 160 X-ray images. Despite the nice methodology flow, conclusions about the results (100% Accuracy) should be carefully done, as the relatively small dataset is not good enough for being split it into testing, training, and validation groups for drawing conclusions.

### 4.4. Critical Analysis for the Selected Papers

The performance results presented in Table 2 show some interesting values. However, we note that there is a good portion of the paper that needed to perform data augmentation as a way to artificially increase the number of samples, especially for the COVID-19 classes. This type of process can lead to an overfitting set of results, meaning that the same samples can be used for the training and testing stages, which leads to an increase in the discrimination rates.

A direct comparison between papers was not possible, even though there are a couple of articles that share the same database. This is due to the use of data augmentation, which was referred to in the previous paragraph, the use of different features, as some were extracted from the pre-trained classifier, and the use of a smaller sample size for the training/testing, making it impossible to know if the same sample was used between papers that used the same database.

## 5. Conclusions

Even though COVID-19 already has different types of vaccines, the quick and accurate detection of the pathology is extremely important for the prevention of worst-case scenarios.

In this paper, we have reviewed a few articles related to three different types of computerized diagnostic support systems: ECG (including Heart Rate Variability), Voice, and X-ray.

We conclude that the computerized detection of COVID-19 already has promising results in the literature, showing that it might be possible to detect the disease without the need for an RT-PCR test. However, there is still room for improvement, given the vast symptomatology and better comprehension of an individual’s evolution of the disease.

### Future Directions

The goals defined for this review were accomplished, however for future directions, it would be interesting to combine a variety of research engines and analyze the existing methodologies for the COVID-19 prognosis.

Another contribution that we believe will bring a huge benefit is the increase of the number of public databases available and that the sample size of those databases should be enlarged, especially for the COVID-19 positive groups. This would make the algorithms already developed more robust, paving the way for their implementation in clinical settings.

## Figures and Tables

**Table 1:** State-of-the-art methods of the present review.

Ref.	Dataset	Data Augmentation	Source	Features	Machine Learning Classifier	Cross-Validation
[17]	ECG images dataset of cardiac and COVID-19 patients (1937 records)	Yes	ECG	Feature extraction from ECGConvnet (transfer learning)	ECGConvnet	Yes
[18]	ECG images dataset of cardiac and COVID-19 patients (1937 records)	No	ECG	Feature extraction from SEResNet18 (transfer learning)	SEResNet18	Yes
[19]	ECG images dataset of cardiac and COVID-19 patients (1937 records)	Yes	ECG	Feature extraction with VGG16 pre-trained (transfer learning)	CNN VGG16	Yes
[20]	ECG images dataset of cardiac and COVID-19 patients (1937 records)	Yes	ECG	ResNet-50, Inception V3, Xception, InceptionResNet and DenseNet-201 pre-tained feature extraction (transfer learning)	ECG-BiCoNet (CNN)	Yes
[21]	ECG images dataset of cardiac and COVID-19 patients (1937 records)	Yes	ECG	Feature extraction from 3D CNN (transfer learning)	3D CNN	Yes
[22]	ECG images dataset of cardiac and COVID-19 patients (1937 records)	Yes	ECG	Feature extraction from InceptionV3 pre-trained (transfer learning)	CNN	Yes
[35]	UdL+UC+Coswara+Virufy+Pertussis (813 samples)	Yes	Voice	Energy, instantaneous frequency, instantaneous frequency peak, Shannon entropy, instantaneous entropy, spectral information entropy, spectral information, and kurtosis	Random Forest	Yes
[36]	Corona Voice Detect project with Voca.ai (3415 samples)	Yes	Voice	Mel frequency cepstral coefficients	XGBoost	Yes
[37]	Crowd-sourced Respiratory Sound Data	Yes	Voice	C-19CC	SVM	Yes
[38]	Coswara + COUGHVID + ComPare-CCS (2026 samples)	Yes	Voice	Log Mel Spectrogram	CNN14	No
[39]	ESC-50 (5435 samples)	No	Voice	Mel frequency cepstral coefficients	Deep Transfer Learning-based Multi Class classifier	Yes
[40]	Coswara database (1027 samples)	No	Voice	Fundamental frequency, jitter and shimmer, harmonic to noise ratio, mel-frequency cepstral coefficients, first and second derivatives of cepstral coefficient, spectral centroid and spectral Roll-off	SVM	No
[41]	Coswara database (909 samples)	No	Voice	Energy, entropies, correlation dimension, detrended fluctuation analysis, Lyapunov Exponent and fractal dimensions	XGBoost	Yes
[46]	Covid chestxray dataset + Chex Pert dataset (5370 samples)	No	X-ray	Feature extraction from Resnet18 pre-trained (transfer learning)	Resnet18	Yes
[47]	Covid chestxray dataset + Chex Pert dataset (5184 samples)	Yes	X-ray	Feature extraction from LetNet-5 (transfer learning)	Extreme Learning Machine	No
[44]	Covid chestxray dataset + Kaggle repository + Open-i repository (160 samples)	Yes	X-ray	Deep feature extraction based on VGG16, ResNet50 and InceptionV3 (transfer learning)	CNN Inceptionv3	No
[48]	Covid chestxray dataset + Labeled Optical Coherence Tomography + Chest X-ray Images for Classification	Yes	X-ray	Feature extraction from CNN (transfer learning)	Modified ResNet-18	No
[49]	Covid chestxray dataset + Kaggle repository (50 samples)	Yes	X-ray	Feature extracted by CNN ResNet50 (transfer learning)	SVM	No
[50]	Covidx Dataset (14,003 samples)	Yes	X-ray	Feature extracted by ResNet-18 (transfer learning)	MSRCovXNet (multi-stage residual network)	Yes
[51]	COVID-19 CHEST X-RAY DATABASE+ COVID-19 Database + COVID-Chestxray Database + ChestX-ray8 + chest-xray-pneumonia (1560 samples)	No	X-ray	Contrast, correlation, energy, entropy, homogeneity, Mittag-Leffler distribution, Pareto distribution, and Cauchy distribution	KNN	No

**Table 2:** State-of-the-art methods that fit on the present review - discrimination rates (N/A: not applicable).

Ref.	Accuracy	F1-Score	Sensitivity	Specificity
[17]	99.74%	99.70%	99.70%	≈100%
[18]	83.17%	85.38%	84.81%	86.28%
[19]	81.39%	N/A	N/A	N/A
[20]	91.73%	91.80%	91.70%	95.90%
[21]	92.00%	92.03%	95.99%	92.00%
[22]	97.83%	97.82%	97.83%	98.86%
[35]	85.53%	85.58%	85.96%	85.09%
[36]	99.00%	69.00%	70.00%	N/A
[37]	85.70%	N/A	N/A	N/A
[38]	88.19%	N/A	N/A	N/A
[39]	92.64%	92.66%	92.64%	97.55%
[40]	97.07%	82.35%	93.33%	97.37%
[41]	98.46%	N/A	N/A	N/A
[46]	≈98%	N/A	N/A	N/A
[47]	98.83%	N/A	N/A	N/A
[44]	100%	100%	100%	100%
[48]	96.73%	N/A	N/A	N/A
[49]	95.38%	95.52%	97.29%	93.47%
[50]	82.20%	N/A	N/A	N/A
[51]	100%	N/A	N/A	N/A

## Data Availability

Not applicable.
